# The emerging use of aromatase inhibitors for endometriosis treatment

**DOI:** 10.1186/1477-7827-9-87

**Published:** 2011-06-21

**Authors:** Warren B Nothnick

**Affiliations:** 1 Departments of Obstetrics and Gynecology and Molecular and Integrative Physiology, University of Kansas Medical Center, 3901 Rainbow Boulevard, Kansas City, KS 66160, USA

## Abstract

Endometriosis is defined as the growth of endometrial tissue outside of the uterine cavity. The disease occurs primarily in women of reproductive age but recurrent endometriosis is also detected in post-menopausal women. Regardless of age, endometriosis is associated with pain and reduces the quality of life for millions of women world-wide. Conventional therapies focus on reducing systemic levels of estrogen which results in cessation of endometriotic implant growth and pain symptoms associated with the disease. However, these treatments are not effective in all women and are not without side effects. Based upon the discovery that endometriotic tissue over-expresses aromatase, an enzyme critical for estrogen production, emphasis has been placed upon the use of aromatase inhibitors for the treatment of endometriosis and its associated symptoms. This article will review the rationale behind the use of aromatase inhibitors in treating endometriosis and summarize those studies which have evaluated the use of aromatase inhibitors in the treatment of endometriosis and its associated symptoms.

## Review

### Aromatase and estrogen biosynthesis

**Figure 1:**
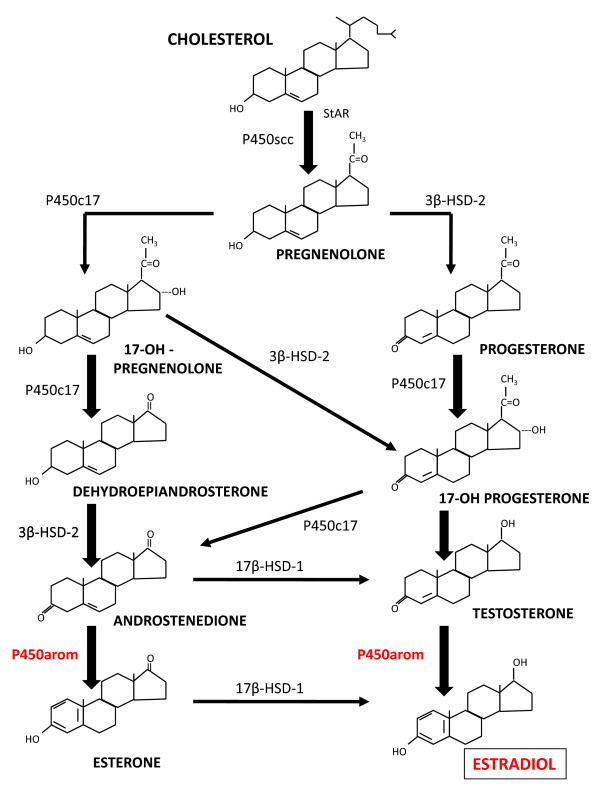
**Steroidogenic pathway leading to the production of estradiol**. Elevated aromatase (P450 arom) expression by endometriotic implant tissue is proposed to lead to the local production of estradiol and subsequent implant growth. P450scc = side chain cleavage enzyme; P450c17 = 17 α-hydroxylase; 3β-HSD = 3β-hydroxysteroid dehydrogenase type 2; 17β-HSD-1 = 17β-hydroxysteroid dehydrogenase type 1.

Estradiol 17β (or estrogen) is the major biochemical driving force for endometriotic implant growth. In women of reproductive age, estrogen is derived primarily from the ovaries and the notion that systemic estrogen drives implant growth has long been considered dogma. However, substantial evidence also points to the endometriotic implant as an intracrine source of estrogen. This locally produced estrogen results from over-expression of P450 aromatase (referred to hence forth as aromatase) by endometriotic tissue (Figure 1). As a result, considerable emphasis has been placed upon the use of aromatase inhibitors to curtail endometriotic implant production of estrogen and subsequent implant growth. The following review highlights the discovery of endometriotic aromatase expression and the use of aromatase inhibitors in the treatment of endometriosis.

### Aromatase expression in endometriotic tissue

The first report describing expression of aromatase in peritoneal endometriotic implants was published in 1996 by Noble and colleagues [1]. Since this initial report, numerous independent investigators have described the expression and cellular localization of aromatase transcript and protein in endometriotic tissue [2-8] as well as eutopic endometrium from women with the disease [2,3,5,8-13]. The majority of these studies demonstrate that aromatase mRNA can be detected in most but not all endometriotic biopsies or eutopic endometrial biopsies from women with endometriosis; however, none of the endometrial biopsies from women without endometriosis expressed aromatase transcript. Within endometriotic implants and eutopic endometrium from women with endometriosis, aromatase transcript expression has been shown to be significantly greater in epithelial cells compared to stromal cells.

Aromatase protein expression has been localized to both epithelial and stromal cells of the endometriotic implant and eutopic endometrium; however, the pattern, and relative level, of expression within each cell type is inconsistent. Epithelial cells do appear to be the major source of endometriotic/endometrial tissue aromatase protein expression.

While the majority of the literature supports the elevated expression of aromatase in endometriotic tissue, a recent report by Colette and colleagues [14] refutes the expression of aromatase in this tissue. In this study, human peritoneal, ovarian and rectovaginal endometriotic implants as well as matched eutopic endometrial biopsies were evaluated for aromatase protein and mRNA expression. In contrast to previous data, the findings from this study suggested that aromatase protein is not expressed in endometriotic tissue or in eutopic endometrium from women with the disease and only low but discernible levels of aromatase transcript were detected in ovarian endometriomas. The authors also raise the possibilities that aromatase transcript expression in ovarian endometriomas may be due to "contaminating" ovarian tissue and that elevated aromatase induction of estrogen production may result from local pelvic cavity tissues such as the peritoneum or adipose.

While this explanation seems plausible for the discrepancy between the study by Colette and colleagues [14] compared to previous studies evaluating aromatase expression in endometriotic or endometrial tissue, a more recent *in vitro *study [15] supports the notion that aromatase is indeed expressed in endometriotic and endometrial cells from women with endometriosis. Using isolated stromal cells from endometriotic chocolate cysts and endometrial biopsies, Izawa and colleagues [15] demonstrated that endometriotic stromal cells secrete estrogen and that this secretion could be increased by addition of testosterone to the media. Further, increased expression of aromatase transcript was confirmed in the endometriotic cell cultures and that this expression may be associated with epigenetic modifications of the aromatase gene. Molecular alterations leading to aberrant aromatase production by endometriotic stromal cells were first reported by Zeitoun and colleagues [16]. Using isolated stromal cells from endometriotic and eutopic endometrial tissue, these investigators demonstrated that the stimulatory transcription factor, SF-1, was over-expressed in endometriotic stromal cells compared to stromal cells from eutopic endometrium. Further, expression of a transcription inhibitory factor, COUP-TF was not expressed in endometriotic stromal cells but was expressed in stromal cells from eutopic endometrium. As both of these factors compete for the same *cis*-acting element, it was proposed that over-expression of SF-1 coupled with the absence of COUP-TF in endometriotic stromal cells leads to expression of aromatase and local estrogen production by endometriotic stromal cells.

In summary, the general consensus is that aromatase transcript and protein are elevated in endometriotic implants with stromal cells being the major source of the aberrant expression. Further, there is a great deal of variability in the expression of aromatase protein in the eutopic endometrium from women with the disease. The finding that endometriotic implant aromatase expression is elevated supports the notion of autonomous estrogen production by this tissue. These findings in turn have lead to the use of aromatase inhibitors in the medical management of endometriosis.

### Aromatase inhibitors in the medical management of endometriosis

#### Anastrozole

Anastrozole is a non-steroidal, competitive aromatase inhibitor which mimics normal enzyme substrate and competes for binding sites on endogenous aromatase enzyme [17]. Marketed under the trade name Arimidex (AstraZeneca), anastrozol has been primary used for treatment of breast cancer after surgery and for metastases in postmenopausal women. As aromatase has been proposed to play a role in the pathogenesis of endometriosis, its use as a means to treat the disease has emerged.

The first report describing the use of an aromatase inhibitor in the treatment of endometriosis was by Takayama and colleagues [18] in 1998. In that study, a 57-year-old woman who presented with recurrent severe endometriosis after hysterectomy and bilateral salpingo-oophorectomy was administered oral anastrozole for 9 months. Anastrozole administration resulted in a significant reduction in pelvic pain as well as lesion size in this patient suggesting that aromatase inhibitors may be successful candidate drugs in the treatment of endometriosis. Additional studies evaluating the efficacy of anastrozole in treatment of endometriosis associated pain have been reported; their findings are discussed below and are summarized in Table 1.

**Table 1:** Successful use of anastrozole in the treatment of endometriosis associated symptoms

*Patient group*	*Summary of Results*	*Reference*
Case report (one post-menopausal patient, recurrent endometriosis)	Rapid decrease in pelvic pain Decrease in implant mass	[18] Takayama *et al.*
Pre-menopausal women (N = 2) (intolerant/failed previous treatment, severe pain symptoms)	Decrease in associated symptoms Decrease in implant mass (1 of 2 patients)	[19] Shippen & West
Pre-menopausal women (N = 15) (refractory endometriosis, pelvic pain)	Decreased pelvic pain, affect on implant mass not determined	[20] Amsterdam *et al*.
Pre-menopausal women (N = 80) (prior conservative surgical therapy; N = 40 goserelin vs. N = 40 goserelin + anastrozole)	Decreased symptom recurrence rate Increased pain-free interval	[21] Soysal *et al*.
Pre-menopausal women (N = 10) (endometriosis resistant to conventional therapy)	Increased quality of life, decreased dyspareunia (9/10 patients), change in implant mass inconclusive	[22] Hefler *et al*.
Pre-menopausal women (N = 3) (endometriosis resistant to conventional therapy)	Decreased pelvic pain, affect on implant mass not determined	[23] Verma & Konje

In 2004, Shippen and West [19] reported treatment of two pre-menopausal women whom had sought surgical intervention for severe endometriosis and pain. Both patients were treated with anastrozole combined with progesterone, calcitriol and rofecoxib for six repeated 28-day cycles. Treatment resulted in a rapid, progressive elimination of symptoms over 3 months with the maintenance of remission of symptoms for over a year after treatment in both cases. Treatment was well-tolerated with no reports of adverse effects. Moreover, pregnancy was achieved after a year in both cases while in one case, a follow-up laparoscopy at 15 months after treatment confirmed the absence of disease.

A larger assessment of the effect of anastrozole in combination with oral contraceptives was conducted by Amsterdam and co-workers [20] in which fifteen premenopausal patients with documented refractory endometriosis and chronic pelvic pain were treated. Women were administered anastrozole (1 mg) combined with ethinyl estradiol (20 μg) and levonorgestrel (0.1 mg) daily for 6 months. Pelvic pain was assessed after each month while side effects, blood counts and bone density were monitored. Significant reduction in pelvic pain scores were noted in 14 of 15 patients and occurred as early as one month after treatment initiation. Treatment side effects were mild and improved over time, but serum estrogen levels were suppressed during treatment. Although the authors did not provide follow-up assessment to determine if and when symptoms returned after completion of therapy, these results do suggest that anastrozole in combination with oral contraceptives may be an effective therapy for premenopausal women with endometriosis which is refractory towards conventional therapies and who do not wish to conceive.

The efficacy of anastrozole in combination with GnRH analogues in treating endometriosis-associated pain has also been conducted [21]. Using a prospective, randomized design, eighty patients were randomized to receive either anastrozole (1 mg/day) plus 3.6 mg of goserelin or placebo plus goserelin every 4 weeks for 24 weeks. Patients were evaluated for pain at the end of the 24 week treatment period and then again at 6, 12, 18 and 24 weeks after the end of medical treatment. Compared to goserelin alone, co-treatment with anastrozole significantly increased the pain-free interval (over 2.4 months compared to 1.7 months) and decreased symptom recurrence rates (7.5% recurrence vs. 35% recurrence). Anastrozole in combination with goserelin suppressed serum estrogen levels as well as significantly reduced greater lumbar spine bone mineral density at the end of the treatment period compare to the goserelin-only group. However, this was not associated with a reduction in the menopausal quality of life, and the bone mineral density lose, although statistically different, was not determined to be of concern.

The use of anastrozole alone has been reported for the treatment of rectovaginal endometriosis [22]. Ten premenopausal women were treated with vaginally administered anastrozole (0.25 mg in a 2 gram vaginal suppository) for 6 months. During active treatment, pain was assessed daily while quality of life was assessed monthly and these assessments continued for 1 month after cessation of treatment to provide post-treatment values. Vaginally administered anastrozole had no effect on pelvic pain, dyspareunia, number of sexual contacts, duration and intensity of menstruation during or after treatment, but dysmenorrhea did improve in all but one patient. Anastrozole administration did result in improvement of quality of life, particularly with respect to physical and social functioning. Also of interest was the finding that serum estrogen levels were not affected by anastrozole administration, which, as pointed out by the authors, may be one reason for the lack of therapeutic efficacy of anastrozole in this study.

An additional, more recent study which evaluated the use of anastrozole alone was conducted by Verma and Konje [23]. Three pre-menopausal patients with refractory endometriosis and chronic pelvic pain were treated with anastrozole for a 6-month period (while one was treated with letrozole) after which pelvic pain, serum estradiol, FSH and LH were assessed in addition to bone density changes. In all patients, anastrozole (as well as letrozole) treatment was associated with a significant reduction in pelvic pain and this occurred independent of changes in systemic estradiol, LH or FSH levels. Bone density scores did not differ after treatment. The major side effect was irregular bleeding. These findings suggest that anastrozole (and letrozole) is beneficial in premenopausal patients with chronic pelvic pain with minimal side effects and that these benefits were not associated with changes in circulating estrogen levels.

#### Letrozole

Like anastrozole, letrozole (trade name Femera [Novartis]) is also a non-steroidal, competitive aromatase inhibitor which has been used primarily for treatment of breast cancer [16]. Letrozole has been used alone or in combination with steroid analogs to treat endometriosis (summarized in Table 2). The first description of letrozole treatment in patients with endometriosis was reported in 2004. In that study [24], 10 premenopausal patients with endometriosis were subjected to an open-label, non-randomized design and were administered letrozole (2.5 mg daily) in combination with the progestin and norethindrone acetate (2.5 mg) for 6 months. Pelvic pain, endometriosis stage, bone density and serum estrogen, LH and FSH were evaluated before and after treatment administration. Administration of letrozole resulted in a significant reduction in pelvic pain and stage of endometriosis and was not associated with changes in serum estrogen levels or bone density.

**Table 2:** Successful use of letrozole in the treatment of endometriosis associated symptoms

*Patient group*	*Summary of Results*	*Reference*
Pre-menopausal women (N = 1) (endometriosis resistant to conventional therapy)	Decreased pelvic pain, affect on implant mass not determined	[23] Verme & Konje
Pre-menopausal women (N = 10) (endometriosis resistant to conventional therapy)	Decrease in pelvic pain Decrease in implant mass	[24] Ailawadi *et al*.
Pre-menopausal women (N = 82) (intolerant/failed previous treatment, severe pain symptoms, N = 37 Letrozole + norethisterone acetate vs. N = 38 Norethisterone acetate alone)	Decrease in pelvic pain and deep dyspareunia, affect on implant mass unknown	[25] Ferrero *et al*.
Pre-menopausal women (N = 2) (bladder endometriosis, non-responsive to conventional therapy, pelvic pain and urinary symptoms)	Decreased pelvic pain and urinary symptoms, affect on implant mass not determined	[26] Ferrero *et al*.
Women with colorectal endometriosis (N = 6; pain and intestinal symptoms)	Decreased pain and intestinal symptoms, affect on implant mass unknown	[27] Ferrero *et al*.
Pre-menopausal women (N = 5) (recurrent ovarian endometriosis, chronic pelvic pain)	Decreased pelvic pain, decreased implant mass	[28] Lall *et al*.
Pre-menopausal women (N = 1) (recurrent endometriosis resistant to conventional therapy, pelvic pain dyspareunia)	Decreased pelvic pain and dyspareunia, affect on implant mass not determined	[29] Razzi *et al*.
Post-menopausal woman (N = 1) (recurrent endometrioma, pain)	Decreased pain, decrease in implant mass	[30] Fatemi *et al*.
Post-menopausal woman (N = 1) (severe pelvic pain)	Decreased pelvic pain, affect on implant mass unknown	[31] Mousa *et al*.
Post-menopausal woman (N = 1) (recurrent abdominal wall endometriosis)	Decreased implant mass	[32] Sasson & Taylor

Ferrero and colleagues [25] conducted a prospective, open-label, non-randomized trial consisting of 82 women with pain symptoms caused by rectovaginal endometriosis. Subjects were administered either letrozole plus norethisterone actetate or norethisterone acetate alone for 6 months. Changes in pain symptoms as well as side effects were monitored during the course of treatment and then again at a 12 month follow-up. At 3 months of treatment, chronic pelvic pain and deep dyspareunia were significantly decreased in both treatment groups compared to baseline values, while at 6 months, patients treated with both letrozole and norethisterone reported significantly less chronic pelvic pain and deep dyspareunia. However, at 6 months post-treatment, pain symptoms recurred in both groups. Study subjects also reported lower patient satisfaction with treatment and a higher percentage of adverse effects (such as weight gain, joint pain, migranes and spotting) in the letrozole group. Patients administered letrozole in combination with norethisterone acetate experienced a greater reduction in pain during active treatment, however, the combination therapy was associated with a higher cost and incidence of unwanted side effects and lower patient satisfaction. Based upon these findings and considering the higher cost of the combination regimen, the authors concluded that aromatase inhibitors should only be administered to patients who previously failed to respond to conventional therapies (such as progestins and/or oral contraceptives) and elect not to have surgical removal of the disease. Accordingly, these benefits and drawbacks should be taken into account when discussing combination letrozole and norethisterone acetate therapy for women with endometriosis-associated pain.

In 2010, 3 additional small scale studies were conducted evaluating the efficacy of letrozole in the treatment of bladder endometriosis [26], colo-rectal endometriosis [27] and recurrent ovarian endometriomas [28]. Two premenopausal patients with bladder endometriosis [26] were treated with letrozole (2.5 mg/day) and norethisterone actetate (2.5 mg/day) for 6 months. Pain and urinary symptoms were markedly improved in both patients. After interruption of the 6 month treatment period, one patient developed myalgia and severe arthralgia and pain and urinary symptoms returned a few months later while the other patient reported no adverse effects. In a separate study [27], 6 women with colo-rectal endometriosis who reported pain and intestinal symptoms were treated with letrozole (2.5 mg/day) and norethisterone acetate (2.5 mg/day) for 6 months. General pain, non-menstrual pelvic pain, deep dyspareunia, dyschezia, intestinal cramping and bloating were improved in all patients, and 4 of the 6 patients indicated that the treatment improved their gastrointestinal symptoms. Letrozole (2.5 mg/day) in combination with desogestrel (0.15 mg) and ethinyl estradiol (0.03 mg) has been reported to significantly improve pelvic pain in women with ovarian endometriomas [28]. Treatment of these patients for 6 months with the combination therapy also induced a complete disappearance of the endometriomas but no change in bone density.

Case reports have also suggested that letrozole either alone [29] or in combination with steroids [29-31] is effective in treatment of endometriosis-associated pelvic pain. Razzi and colleagues [29] reported that a 31 year old woman with recurrent endometriosis after subtotal hysterectomy with bilateral oophorectomy who was administered letrozole alone (2.5 mg) showed significant reduction in pelvic pain and dyspareunia during the 6 months of treatment with no adverse impact on bone density. Similar positive outcomes using letrozole in combination with progestins were noted in a post-menopausal patient with recurrent endometrioma [30] and in a middle-aged woman with endometriosis and severe pelvic pain after hysterectomy and bilateral salpingo-oophorectomy [31]. More recently, Sasson and Taylor [32] reported the case of a post-menopausal woman with a large, recurrent abdominal wall endometrioma who was successfully treated with letrozole (5 mg) and medroxyprogesterone acetate.

While the majority of the literature suggests that letrozole may be effective in treating patients with endometriosis, not all clinical trials using letrozole were successful. An open-label prospective study in 2007 [33] reported that letrozole (2.5 mg/day) combined with norethisterone acetate (2.5 mg/day) quickly reduced the intensity of symptoms related to the presence of rectovaginal endometriosis. However, pain recurred at 3-month follow-up. Five of the initial 12 women treated, underwent surgery during the follow-up, and histological examination of rectovaginal nodules revealed the presence of active endometriotic lesions. In another open-label prospective study [34], 12 women with stage IV refractory endometriosis were treated with letrozole (2.5 mg) and desogestrel (75 μg). Unfortunately, all 12 patients developed ovarian cysts and were unable to complete the scheduled six-month treatment regime. However, at the interruption of treatment, all women reported significant improvements in dysmenorrhea and dyspareunia, but pain symptoms quickly recurred at 3-month follow up.

### Aromatase inhibitors and fertility restoration in women with endometriosis

The majority of studies and trials evaluating the use of aromatase inhibitors in women with endometriosis have focused on relief of pelvic pain and/or effect on endometriotic implant size/severity of disease. However, one recent pilot study conducted by Lossl and colleagues [35] concurrently evaluated the effect of anastrazole (coupled with goserelin) on endometriomal volume, CA125 levels and standard IVF (in vitro fertilization) fertility parameters. Twenty women with endometriosis undergoing IVF/ICSI were treated with goserelin (3.6 mg s.c.) on treatment days 1, 28 and 56 and anastrazole (1 mg daily) from days 1 to 69 of the study, with ovarian stimulation occurring on day 70. Both endometriomal volume and CA125 levels (as a marker of endometrioma activity) decreased by 29% and 61%, respectively during the combined down-regulation. The average number of oocytes retrieved was 7.5 and the fertilization rate was 78%. Nine of the twenty patients conceived while five of these had clinical pregnancies with three of them delivering healthy offspring. This study demonstrated that combined anastrazole and goserelin down-regulation markedly reduces endometriomal volume and disease activity which is compatible with pregnancy and delivery. Concern was expressed with the rate of pregnancy loss (from 5 chemical pregnancies to 3 patients delivering) but it should be emphasized that larger, controlled studies must be conducted to further evaluate this initial observation. This contention is supported by a recent report [36] in which 159 infertile women undergoing controlled ovarian stimulation and artificial insemination treated with the aromatase inhibitor, letrozole in combination with FSH, exhibited comparable pregnancy rates with less cancelled cycles and less FSH required for stimulation compared to FSH-treated patients alone.

### Use of aromatase inhibitors in animal models of endometriosis

In addition to human trials, animal studies have also evaluated aromatase inhibitors in the treatment of endometriosis and support the notion that inhibition of endometriotic implant aromatase activity leads to reduction in endometriotic implant mass. Aromatase has been shown to be expressed in endometriotic implants of mice with experimentally-induced endometriosis [37]. Mice with experimentally-induced endometriosis in which the expression of aromatase has been genetically disrupted exhibit significantly smaller endometriotic implants [38] validating the use of such animal models.

In a side by side comparison [37], both anastrozole and letrozole significantly decreased endometriotic implant size in mice with experimentally-induced endometriosis. Both aromatase inhibitors decreased cell proliferation, but the impact on cell apoptosis was time-dependent with respect to time of intervention after disease establishment. Lastly, both anastrozole and letrozole decreased peritoneal fluid vascular endothelial cell growth factor levels while only anastrozole decreased peritoneal fluid PGE levels.

The effect of anastrozole on experimental endometriosis in rats has also been evaluated. Alintas and colleagues [39] conducted a randomized, placebo-controlled, single-blind study comparing the effect of anastrozole and raloxifene on endometriotic implant volume (size) after 8 weeks of treatment. The authors found that, compared to placebo, both anastrozole and raloxifene were found to be equally effective in reducing endometriotic implant volume. No difference was detected in implant size between the two drugs.

The efficacy of letrozole in endometriosis treatment has also been examined in rats with surgically-induced endometriosis [40]. In this study by Oner and colleagues, rats with surgically-induced endometriosis were randomly assigned to receive either one of two doses of metformin (100 or 200 mg/kg/day), letrozole (0.1 mg/kg/day) or placebo for 4 weeks after which endometriotic implant size and pelvic adhesions were determined. Metformin (both doses) and letrozole caused a significant reduction in endometriotic implant size. While letrozole did not affect pelvic adhesion, metformin reduced the severity of pelvic adhesions irrespective of dose.

Collectively, these studies demonstrate that letrozole and anastrozole are effective in reducing endometriotic implant size in rodents with experimentally-induced endometriosis. The use of these and other genetically modified mice (tissue-specific aromatase knockout or over-expression) coupled with the use of aromatase inhibitors should allow for a thorough dissection of the mechanisms by which these inhibitors work to induced endometriotic implant regression.

## Conclusions

The majority of the evidence in the literature supports the notion that aromatase expression is elevated in human endometriotic tissue, and this concept is further supported in studies using animal models for the disease. Based upon this expression, it has been proposed that elevated aromatase activity in endometriotic tissue leads to local estrogen production and endometriotic lesion growth which is associated with disease symptoms such as pelvic pain. Not surprising, reduction of aromatase activity by aromatase inhibitors has been associated with reduced endometriotic lesion size in both animal models and in human studies. In human subjects, aromatase alone, or in combination with steroids, appears to be effective in reduction of endometriosis-associated symptoms such as pelvic pain. Aromatase inhibitors appear to be most beneficial in the treatment of endometriosis in women with recurrent endometriosis who have not had success with more conventional treatment regimes such as gonadtropin releasing agonists/antagonists or steroidal analogues. However, one must keep in mind that aromatase inhibitors exhibit suboptimal tolerability and greater costs compared to some of the more conventional therapies. Clearly, aromatase inhibitor therapy may have a place in endometriosis treatment of a subset of patients suffering from the disease and benefits and limitations of these compounds must be discussed with patients. Future effort should be directed towards performing larger, multi-center trials with aromatase inhibitors to provide a more robust assessment of the efficacy of these compounds in the treatment of endometriosis and its associated symptoms.

## Competing interests

The author declares that they have no competing interests.
